# One-Pot Detection of miRNA by Dual Rolling Circle Amplification at Ambient Temperature with High Specificity and Sensitivity

**DOI:** 10.3390/bios15050317

**Published:** 2025-05-15

**Authors:** Wenhua Sun, Kunling Hu, Ziting Song, Ran An, Xingguo Liang

**Affiliations:** 1 State Key Laboratory of Marine Food Processing & Safety Control, College of Food Science and Engineering, Ocean University of China, No. 1299 Sansha Road, Qingdao 266404, China; sunwenhua4988@stu.ouc.edu.cn (W.S.); klhu1@gzu.edu.cn (K.H.); szt@stu.ouc.edu.cn (Z.S.); 2 Laboratory for Marine Drugs and Bioproducts, Qingdao Marine Science and Technology Center, No. 1 Wenhai Road, Qingdao 266237, China

**Keywords:** dual RCA, microRNA detection, ssDNA circularization, circular ssDNA, splint

## Abstract

Rolling circle amplification (RCA) at ambient temperature is prone to false positive signals during nucleic acid detection, which makes it challenging to establish an efficient RCA detection method. The false positive signals are primarily caused by binding of non-target nucleic acids to the circular single-stranded template, leading to non-specific amplification. Here, we present an RCA method for miRNA detection at 37 °C using two circular ssDNAs, each of which is formed by ligating the intramolecularly formed nick (without any splint) in a secondary structure. The specific target recognition is realized by utilizing low concentrations (0.1 nM) of circular ssDNA1 (C1). A phosphorothioate modification is present at G*AATTC on C1 to generate a nick for primer extension during the primer self-generated rolling circle amplification (PG-RCA). The fragmented amplification products are used as primers for the following RCA that serves as signal amplification using circular ssDNA2 (C2). Notably, the absence of splints and the low concentration of C1 significantly inhibits non-target binding, thus minimizing false positive signals. A high concentration (10 nM) of C2 is used to carry out linear rolling circle amplification (LRCA), which is highly specific. This strategy demonstrates a good linear response to 0.01–100 pM of miRNA with a detection limit of 7.76 fM (miR-155). Moreover, it can distinguish single-nucleotide mismatch in the target miRNA, enabling the rapid one-pot detection of miRNA at 37 °C. Accordingly, this method performs with high specificity and sensitivity. This approach is suitable for clinical serum sample analysis and offers a strategy for developing specific biosensors and diagnostic tools.

## 1. Introduction

The rolling circle amplification (RCA) technique has undergone extensive research since its development in the 1990s due to its advantage of isothermal amplification [1]. However, its application has been limited to using targets as templates for circularization to generate a padlock probe, making it difficult to improve the sensitivity of detection. On the other hand, hyper-branched rolling circle amplification (HRCA) [2], a cascade amplification involving multiple hybridizations, primer extension, and strand displacement using two primers, generally requires *Bst* DNA polymerase to carry out amplification at temperatures above 60 °C, making it difficult to achieve high sensitivity and specificity below 37 °C, despite many efforts.

On the other hand, the detection of miRNA (a class of non-coding RNA, ranging from 19 to 25 nucleotides in length) is essential in the fields of biological and medical research [3]. miRNA plays an important role in gene regulation, disease diagnosis, and drug development. For example, the binding of miRNA promotes mRNA degradation and plays a crucial role in post-transcriptional gene regulation [4]. For some diseases, the expression of corresponding miRNA becomes more or less, and an increasing number of studies show the potential of miRNA as a diagnostic biomarker [5,6]. Among them, miRNA-155 is a well-studied miRNA, which has been shown to be associated with various cancers and diseases, such as breast cancer [7], asthma [8], and diabetes [9]. However, the high-sensitivity detection of miRNA remains challenging due to its short sequence length, high homology within families, and low sample abundance. There are various methods for detecting miRNA, such as Northern blotting [10] and reverse transcription quantitative polymerase chain reaction (RT-qPCR) [11,12]. However, applications of these methods are limited by the need for special equipment. Moreover, these methods are complex, time-consuming, and expensive, which means they are not suitable for rapid miRNA detection. Various isothermal nucleic acid amplification techniques have been developed, such as rolling circle amplification (RCA) [13], loop-mediated isothermal amplification (LAMP) [14], recombinase polymerase amplification (RPA) [15], and exponential amplification reaction (EXPAR) [16]. Among them, RCA has been widely applied in biology, medicine, and food safety testing due to its excellent biocompatibility and programmability [17]. Several miRNA detection techniques combining RCA with other methods have been developed, such as RCA-LAMP for detecting let-7a [18], RCA-CRISPR/Cas9 for detecting miR-21 [19], RCA-Cas12a for detecting miR-10b and miR-21 [20], and RCA-APE1 for detecting miR-206 [21]. Although these methods improve the sensitivity and specificity of RCA, they require integration with other technologies and involve complex designs and procedures.

**Scheme 1:**
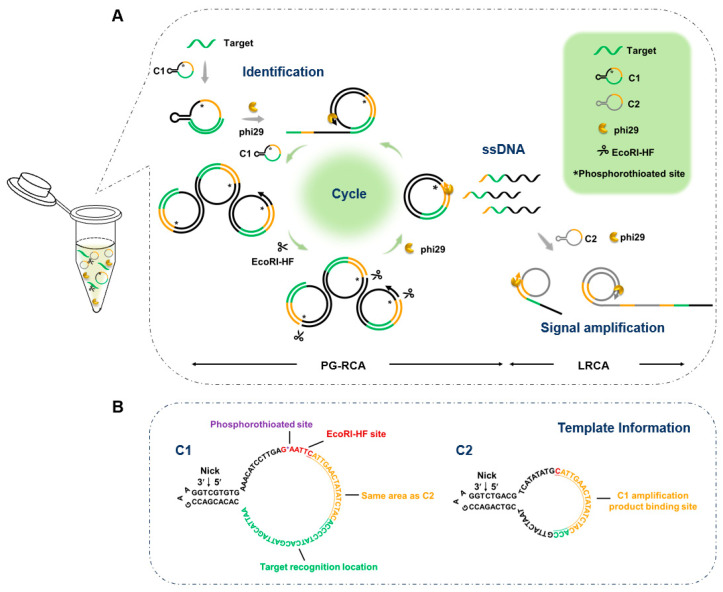
Schematic diagram for the dual RCA strategy (**A**), and the example for detecting the miRNA of miR-155 (**B**). The phosphate bond between G and A in the recognition site (GAATTC) of EcoRI-HF (a restriction enzyme) is replaced by phosphorothioate to avoid its cleavage. The prepared circular ssDNA samples do not involve a splint which can serve as a primer for further RCA. The green line represents the complementary region of the target, and the yellow line represents the extension of the target to the EcoRI-HF enzyme cleavage site region.

In this study, we developed a dual RCA strategy for miRNA detection, achieving high sensitivity at 37 °C without integration with other techniques (Scheme 1). A low concentration (0.1 nM) of circular ssDNA1 (C1) for efficient exponential amplification can avoid non-specific priming, while a high concentration (10 nM) of circular ssDNA2 (C2) for the following LRCA helps to distinguish non-specific amplification. Exponential amplification is achieved by using a modified PG-RCA (primer-self generated rolling circle amplification), in which a phosphorothioate modification on C1 prevents digestion by the restriction enzyme EcoRI-HF (instead of a nicking enzyme required for PG-RCA). This is a challenge to directly use prepared circular ssDNA instead of forming the padlock probe, and the non-specific amplification caused by splint (as the primer) is avoided by using our newly developed splint-free method for ssDNA circularization [22]. Our versatile approach can provide a powerful tool for rapid, one-pot detection under ambient conditions. It does not require thermal cycling instruments, reduces equipment costs, and facilitates the development of point-of-care testing (POCT) devices. Meanwhile, the low concentration self-cyclization template strategy provides conceptual guidance for optimizing other RCA technologies, while enabling the development of additional RCA-based applications.

## 2. Materials and Methods

### 2.1. Materials

The oligonucleotides utilized in this study (detailed in Table S1) and dNTP mixture were commercially acquired from Shanghai Sangon Biotech (Shanghai, China). Multiple enzymatic reagents were procured from specialized manufacturers: Thermo Scientific (Pittsburgh, PA, USA) supplied T4 DNA ligase with its corresponding 10× reaction buffer; ABclonal (Wuhan, China) provided Exonuclease I, Exonuclease III, and phi29 DNA polymerase, accompanied by its 10× buffer system. EcoRI-HF and its optimized 10× buffer were sourced from New England Biolabs (Ipswich, MA, USA). SYBR Green II was obtained from TaKaRa (Beijing, China), while Proteinase K was acquired from TIANGEN (Beijing, China). All remaining chemical compounds and materials were purchased through Sigma-Aldrich (St. Louis, MO, USA).

### 2.2. Preparation of ssDNA Rings

Before circularization, a phosphorylation modification was performed on the 5′-termini of linear ssDNA through enzymatic catalysis with T4 Polynucleotide Kinase (Thermo Scientific, Pittsburgh, PA, USA). The linear phosphorylated ssDNA, T4 DNA ligase, and corresponding buffer are mixed to form the circularization system containing 5 μM ssDNA and 0.25 U/μL T4 DNA ligase in 0.1× T4 ligase buffer (4 mM Tris-HCl, 1 mM MgCl_2_, 1 mM DTT, 0.05 mM ATP, pH7.8@25 °C). The ligation was carried out under 25 °C for 6 h. The reaction was terminated by incubating at 65 °C for 10 min. The circular ssDNA was verified via enzymatic resistance assay using Exonucleases I (1.0 U/μL) and Exonucleases III (1.0 U/μL) at 37 °C for 2 h. Structural validation was conducted through electrophoretic separation on 12% denatured polyacrylamide gel (12% dPAGE).

### 2.3. A Dual RCA for Detection of miRNA

First, a mixture was made containing 0.1 nM C1, 10 nM C2, 400 μM each dNTP, 1× SYBR Green II, 0.5× phi29 buffer (25 mM Tris-HCl, 5 mM (NH_4_)_2_SO_4_, 5 mM MgCl_2_, 2 mM DTT, pH7.5@25 °C), 0.5× rCutsmart buffer (25 mM Potassium Acetate, 10 mM Tris-acetate, 5 mM Magnesium Acetate, 50 µg/mL Recombinant Albumin pH7.9@25 °C), and target miRNA. After heating at 90 °C for 3 min, the solution was cooled at a rate of 0.1 °C/s to 25 °C and left at 25 °C for 5 min. Then, 25 U/mL phi29 DNA polymerase and 50 U/mL EcoRI-HF were added to the reaction mixture and incubated at 37 °C for 120 min. Fluorescence was detected by a Thermo Scientific PikoReal Real-Time PCR System (Type: 5100, Thermo Scientific, USA).

### 2.4. Detection of Serum Samples

The serum samples from a healthy human were supplied by the Chengyang People’s Hospital (Qingdao, China). The serum sample was centrifuged at 10,000 rpm for 15 min at 4 °C. The supernatant was collected and diluted 50 times in a solution containing 0.5× rCutsmart buffer and 0.5× phi29 buffer. Then, different concentrations of miR-155-5p and 4 mg/mL Proteinase K were added. The reaction mixture was incubated at 37 °C for 1 h, and the activity of Proteinase K was terminated by incubating the mixture at 75 °C for 15 min. Finally, the serum sample was added to the solution for dual RCA.

## 3. Results and Discussion

### 3.1. The Strategy of Dual RCA for Efficient Detection of miRNA

The strategy of dual RCA, consisting of PG-RCA (using C1 as the template) and LRCA (using C2 as the template), is illustrated in Scheme 1. A part (23 nt) of C1 (78 nt) is fully complementary to the target miRNA (23 nt), and phosphorothioate modification at the restriction endonuclease site (G*AATTC) protects C1 from cleavage by the restriction endonuclease EcoRI-HF. Phosphorothioate modification was developed by other groups for DNA amplification [23,24]. The RCA using C1 as the template results in the generation of short ssDNA products (78 nt or its multiples) that are completely complementary to C1 and partly (20 nt) complementary to C2 (59 nt). These short ssDNA products serve as primers for C1 to initiate PG-RCA (specific amplification). They also act as primers for C2 to trigger LRCA (signal amplification). A key point for this design is that the concentration of C1 should be low enough (e.g., 0.1 nM), which reduces non-specific binding to C1 to inhibit false positive signals, enabling the highly sensitive and specific detection of miRNA.

### 3.2. Highly Pure Circular ssDNA and Low Concentration Template Significantly Suppress the False-Positive Signals

**Figure 1:**
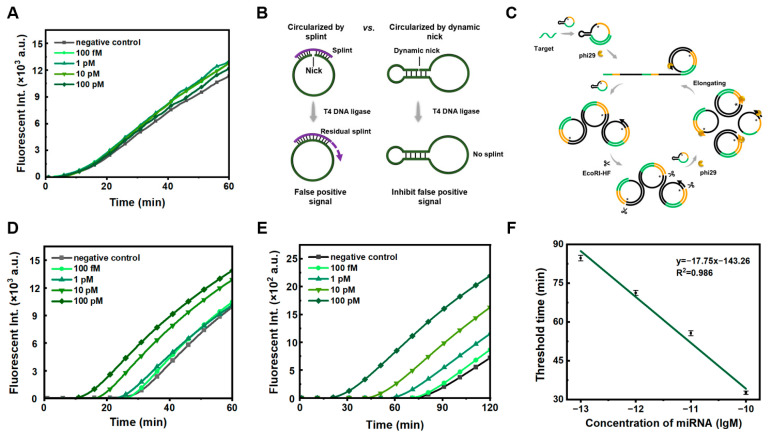
Comparison of PG-RCA between two methods for circularization of circular ssDNA templates. (**A**) Real-time record of PG-RCA at various concentrations of miR-155 using splint-aided circularization. (**B**) Schematic diagrams of two (splint-aided or splint-free) approaches for template circularization. (**C**) The mechanism of PG-RCA by using restriction enzymes (circular ssDNA is prepared by splint-free approach). “*” represents phosphorothioate modification. The green line represents the complementary region of the target, and the yellow line represents the extension of the target to the EcoRI-HF enzyme cleavage site region. (**D**,**E**) Real-time record of PG-RCA at various concentrations of miR-155 using splint-free circularization. PG-RCA conditions: 1 nM C1 for (**B**,**D**) or 0.1 nM for (**E**), 25 U/mL phi29, 50 U/mL EcoRI-HF, 1× SYBR Green II, 0.5× phi29 buffer, 0.5× rCutsmart buffer, 400 μM each dNTP, and incubation at 37 °C. (**F**) Calibration curve showing the linear relationship between the threshold time (Tt) and the logarithmic concentration (lgM) of miR-155, within the range of 10^−13^ to 10^−10^ M. Tt is the threshold time for amplification, defined as the time when fluorescent intensity exceeds the calculated threshold, like Ct values for RT-PCR.

As the splint (used for traditional enzymatic circularization of ssDNA) is fully complementary to the prepared circular ssDNA, the splint can cause false positive signals in PG-RCA if not cleaved completely (Figure 1). As shown in Figure 1A, when a splint is used to circularize the RCA template of C1 (1.0 nM), false positive signals were observed, even after the splint was attempted to be removed using exonucleases (both Exonucleases I and Exonucleases III). As shown in Figure 1C, the RCA products are cleaved simultaneously after amplification, and the cleaved fragments can bind to other C1 templates for further RCA. The circular ssDNA template has to be very pure to successfully realize our strategy.

We developed a method for preparing circular ssDNA by forming a dynamic nick, followed by enzymatic circularization [22]. Unlike the splint-assisted circularization method, the dynamic nick approach uses the linear ssDNA substrate’s own secondary structure for ligation (Figure 1B). The template prepared by forming the dynamic nick has high purity and is free of by-products (Figure S1). When we performed PG-RCA with the newly prepared circular template, false positive signals were significantly reduced (Figure 1D). This indicates that highly pure circular C1 is required for effectively suppressing non-specific amplification. To further suppress false positive signals, we reduced the concentration of the ssDNA ring template from 1 nM (Figure 1D) to 0.1 nM (Figure 1E). Interestingly, non-specific amplification was inhibited to some extent, and the limit of detection was 91.2 fM (Figure 1E,F, using the calculation method reported by Murakami [25]). For comparison, when we reduced the concentration of circular C1 prepared using the splint, non-specific amplification could not be inhibited well (Figure S2). Obviously, this can confirm that the modified PG-RCA using phi29 DNA polymerase and EcoRI-HF is exponential and highly efficient. Accordingly, the preparation of circular ssDNA using a splint-free approach and the utilization of a low concentration of circular ssDNA are essential to significantly suppress false positive signals.

### 3.3. The Dual RCA for Highly Sensitive miRNA Detection and Its Optimization

**Figure 2:**
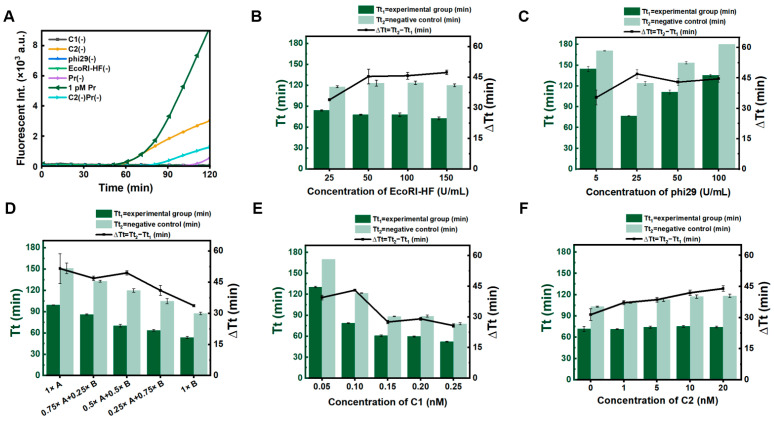
Optimization of conditions for dual RCA. (**A**) Efficiency of dual RCA compared with various controls. The label of (-) indicates the corresponding component is absent. (**B**) Effect of EcoRI-HF concentration on the threshold time (Tt) values. The dark green bars represent the Tt values for the 1 pM target group, while the light green bars correspond to the Tt values for the negative control group. The line graph illustrates the difference in Tt values between the target group and the negative control group. (**C**) Effect of phi29 concentration on Tt values. (**D**) Impact of various buffers on Tt values. Buffer A corresponds to the phi29 buffer, while buffer B corresponds to the rCutsmart buffer. (**E**) Effect of C1 concentration on Tt values. (**F**) Effect of C2 concentration on Tt values.

To improve the sensitivity of miRNA detection, we proposed the dual RCA approach, in which RCA on C1 is used to improve the sensitivity and specificity, and RCA on C2 is used to amplify the signal (Scheme 1). As shown in Figure 2A, fluorescence values (reflecting the amount of amplified DNA) for dual RCA (the green line) were significantly higher than those for C1 alone (the orange line, C2(-)). Interestingly, the signal was markedly weaker for the negative control in which C1 and C2 were present (the purple line), compared to the negative control in which C2 was absent (the light blue line). This indicates that the presence of C2 can reduce false positive signals (Figure S3). Although the dual RCA method introduces some complexity to the system due to the use of two circular ssDNA templates, the addition of C2 significantly suppresses false positive signals and enhances the detection sensitivity, which is particularly crucial for detecting single-stranded nucleic acids such as miRNA.

Subsequently, the concentrations of EcoRI-HF, phi29 DNA polymerase, the buffer, and C1 and C2 were optimized to improve the sensitivity and specificity of dual RCA. As shown in Figure 2B, as the amount of EcoRI-HF increased, Tt_1_ (threshold time for 1 pM miR-155, defined as the time when fluorescent intensity exceeds the calculated threshold, like Ct values for RT-PCR) did not change greatly (only decreased somewhat), and Tt_2_ (the threshold time for the negative control in the absence of primer) increased slightly from 25 U/mL to 50 U/mL of EcoRI-HF, and then remained unchanged. Obviously, ∆Tt (Tt_2_ − Tt_1_), which reflects the specificity of detection, was highest at 50 U/mL of EcoRI-HF, indicating that the 50 U/mL of Eco-RI-HF is sufficient.

Following similar methodology, the concentration of phi29 DNA polymerase was also optimized (Figure 2C). Interestingly, as phi29 concentration increased, Tt for both experimental and control groups first decreased and then increased. This phenomenon may be primarily due to the 3′→5′ exonuclease activity of phi29. It has been reported that phi29 possesses strong 3′→5′ exonuclease activity [26,27]. We found that the amplification efficiency of the primer with phosphorothioate modifications at the 3′ end (PS-Pr) was significantly higher than the primer without modification (Figure S4). Therefore, when the concentration of phi29 is high, stronger exonuclease activity can destroy the produced ssDNA, leading to a decrease in amplification efficiency. Accordingly, we selected 25 U/mL of phi29 for dual RCA.

Because EcoRI-HF (1× buffer B, rCutsmart buffer) and phi29 DNA polymerase (1× buffer A, phi29 buffer) have different buffer requirements, the effects of the buffer ratios and concentrations were investigated (Figure 2D). Although ∆Tt is highest in the 1× buffer A (without buffer B), the reaction time becomes longer, probably due to the higher activity of phi29 and the lower activity of EcoRI-HF under this condition. We ultimately used a mixture of 0.5× buffer A and 0.5× buffer B. In addition, we investigated the concentrations of C1 and C2 (Figure 2E,F). As C1 concentration increased, Tt_2_ gradually decreased, and the ∆Tt value peaked at 0.1 nM C1. As shown in Figure 2F, ∆Tt increased with increasing C2 concentration. This further proves that the addition of C2 can suppress false positive signals. However, excessively high concentrations of C2 can also easily trigger false positive signals by LRCA, so we selected 10 nM of C2.

### 3.4. Sensitivity and Universality of Dual RCA for Detecting miRNA

**Figure 3:**
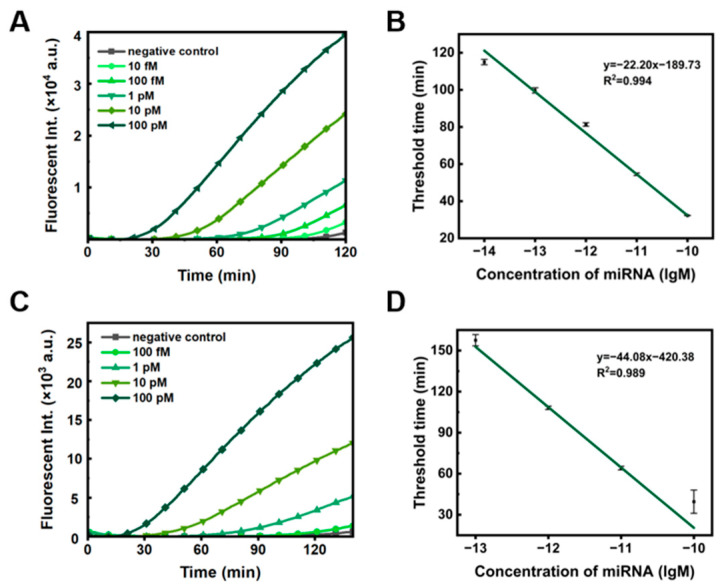
Sensitivity of the dual ssDNA ring system for miRNA detection. (**A**) Fluorescence intensity at various concentrations of miR-155. (**B**) Calibration curve showing the linear relationship between the threshold time (Tt) and the logarithmic concentration (lgM) of miR-155, within the range of 10^−14^ to 10^−10^ M. (**C**) Fluorescence intensity at various concentrations of miR-106. (**D**) Calibration curve depicting the linear correlation between Tt and lgM for miR-106, within the range of 10^−13^ to 10^−10^ M. Other reaction conditions: 0.1 nM C1, 10 nM C2, 25 U/mL phi29, 50 U/mL EcoRI-HF, 1× SYBR Green II, 0.5× phi29 buffer, 0.5× rCutsmart buffer, 400 μM each dNTP, and 37 °C.

Under optimal system conditions, we investigated the sensitivity of dual RCA for detecting miRNA-155. As the concentration of miR-155 increased, the Tt value gradually decreased (Figure 3A), showing a linear relationship with the logarithm of miR-155 concentration ranging from 10^−14^ to 10^−10^ M (Figure 3B, R^2^ = 0.994), and the limit of detection was 7.76 fM. To demonstrate the method’s universality, we designed another two ssDNA circles for dual RCA targeting miR-106 (Figure S5). As expected, the Tt value decreased with increasing miR-106 concentration (Figure 3C), and the limit of detection was 21.88 fM (Figure 3D). Previous studies have shown that miRNA concentrations in human plasma and serum are typically in the sub-picomolar range [28,29]. Thus, the dual ssDNA ring system demonstrates sufficient sensitivity and versatility for miRNA detection. It is noteworthy that only single-stranded nucleic acids can be detected. In addition to miRNA, if we know a part of the sequence (longer than 20 nt) of longer RNAs (e.g., mRNAs, lnc RNAs), this method can also be used to detect them after site-specific cleavage.

### 3.5. The Specificity of Dual RCA

**Figure 4:**
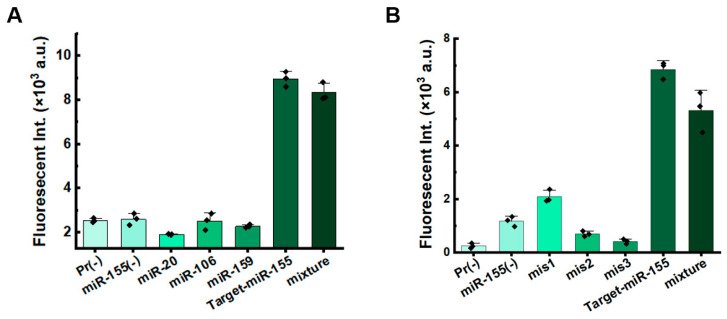
Sensitivity evaluation of specificity for dual RCA. (**A**) Fluorescence intensity for targeting miR-155 in the presence of various other miRNAs. Pr (-) denotes the absence of miRNA; miR-155 (-) indicates the presence of 1 pM miR-20, miR-106, and miR-159, but not target-miR-155; and “mixture” refers to a reaction containing all miRNAs. (**B**) Fluorescence intensity of miR-155 with various numbers of mismatches. Pr (-) denotes the absence of miRNA; miR-155 (-) indicates the presence of 1 pM of mis1, mis2, and mis3, but not target-miR-155; and “mixture” refers to a reaction containing all the miRNAs. Other conditions: 0.1 nM C1, 10 nM C2, 1.0 pM miRNA, 25 U/mL phi29, 50 U/mL EcoRI-HF, 1× SYBR Green II, 0.5× phi 29 buffer, 0.5× rCutsmart buffer, 400 μM each dNTP, and incubation at 37 °C for 120 min.

It is acknowledged that miRNAs exhibit a high degree of familial homology [30,31]. Therefore, detecting miRNAs requires methods with high specificity. We assessed the anti-interference ability of the system for miRNA detection. When non-target miR-20, miR-106, and miR-159 were added to the system targeting miR-155, significant fluorescence signals were detected only in the presence of the target miRNA-155 (Figure 4A and Figure S6A). Furthermore, when non-target miRNAs (miR-155, miR-159, and miR-20) were added to the system targeting miR-106, significant fluorescence signals were detected only in the presence of miRNA-106 (Figure S7). We also tested the specificity using miRNAs with mismatches (Table S1). Compared to the target miR-155, fluorescence signal of sequences with mismatches significantly decreased. For sequences containing a single mismatch, signals were slightly higher than those with double or triple mismatches (Figure 4B and Figure S6B). These results indicate that the dual RCA strategy exhibits excellent selectivity and specificity, demonstrating its potential for practical applications.

### 3.6. Application in Human Serum Analysis

To evaluate the accuracy of the dual ssDNA ring system in real samples, we added various concentrations of miR-155 into 2% human serum for analysis. miRNAs have been shown to exhibit exceptional stability in plasma and serum samples [32], and they can withstand adverse physiological conditions, such as multiple freeze–thaw cycles, long-term storage, and changes in heat and pH values [33,34]. Prior to the detection, serum samples containing various concentrations of miR-155 were pretreated to minimize protein interference. Proteinase K treatment was applied to reduce protein interference in serum samples containing different concentrations of miR-155. As the concentration of miR-155 increased, the Tt value decreased, indicating faster amplification (Figure S8A). The Tt value also exhibited a linear relationship with the logarithm of miR-155 concentrations in the serum sample (Figure S8B, R^2^ = 0.999). The recovery rates of miR-155 at concentrations of 100 fM, 1 pM, 10 pM, and 100 pM were within the range of 87.9–115%, with a precision (expressed as the relative standard deviation, RSD) ranging from 2.51 to 8.13% (Table 1). These results indicate that the dual ssDNA ring system demonstrates significant potential for detecting real biological samples.

## 4. Conclusions

In summary, a dual RCA approach has been established for miRNA detection at 37 °C, including an exponential RCA (using probe C1) for high sensitivity and high specificity, and a linear RCA (using C2) for signal detection. The efficient exponential amplification is realized by using phosphorothioate modification at restriction endonuclease site. The high specificity and sensitivity can be explained as follows: the low-concentration probe C1 (0.1 nM) with high purity specifically recognizes the target miRNA and initiates exponential amplification, generating ssDNA products as primers for both C1 and C2. The interference from non-specific priming is effectively inhibited to decrease false positive signals. The dual RCA approach offers a detection limit of 7.76 fM for miR-155, and enables simple, efficient, and cost-effective miRNA detection without the need for integration with other amplification methods. Additionally, the method is suitable for detecting other nucleic acids that can be used as primers for DNA polymerization, demonstrating promising potential for clinical applications.

## Figures and Tables

**Table 1:** Determination of miRNA in 2% human serum ($\bar{x}$ ± *s*, *n* = 3).

Add (pM)	Found (pM)	Recovery (%)	RSD (%) (*n* = 3)	F	*p*-Value
100	100.9 ± 3.5	100.9	3.45	2228	<0.001
10	10.33 ± 0.84	103.3	8.13		
1	0.879 ± 0.051	87.9	5.80		
0.1	0.115 ± 0.003	115	2.51		

## Data Availability

The data presented in this study are available on request from the corresponding author.

## Supplementary Materials

The following supporting information can be downloaded at: https://www.mdpi.com/article/10.3390/bios15050317/s1. Table S1: The sequences of DNA and RNA used in this study; Figure S1: Circularization results of templates; Figure S2: Real-time record of PG-RCA with splint-aided circularization; Figure S3: Effect of C2 on non-specific amplification; Figure S4: Effect of the 3′→5′ exonuclease activity of phi29 on amplification; Figure S5: Sequence design for detecting miR-106 with dual RCA; Figure S6: Specificity evaluation of dual RCA (miR-155); Figure S7: Specificity evaluation of dual RCA (miR-106); Figure S8: Determination of miRNA in 2% human serum.
