# Supplementary figures and images for: One-Pot Detection of miRNA by Dual Rolling Circle Amplification at Ambient Temperature with High Specificity and Sensitivity
Source: Biosensors (Basel). 2025 May 15;15(5):317. doi: 10.3390/bios15050317 (PMC12109915; doi:10.3390/bios15050317)

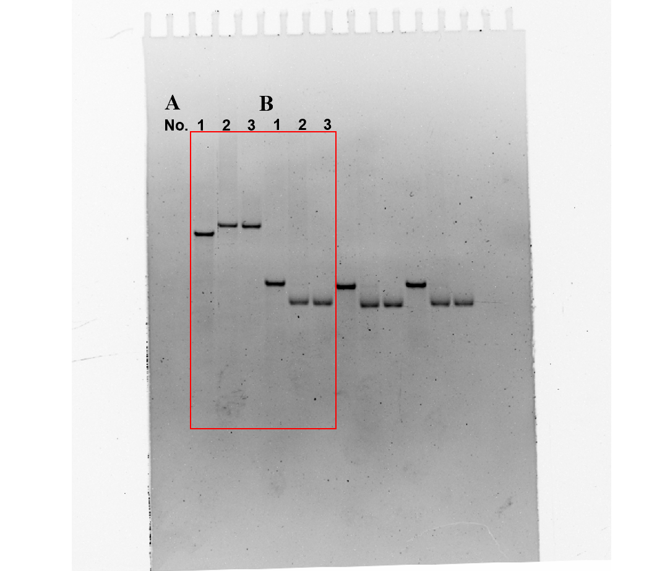

Supplement: Supplementary file 1 [file biosensors-15-00317-s001.zip › Supplementary Files/origin image/Figure S1.tif]
