# Supplementary material for: One-Pot Detection of miRNA by Dual Rolling Circle Amplification at Ambient Temperature with High Specificity and Sensitivity
Source: Biosensors (Basel). 2025 May 15;15(5):317. doi: 10.3390/bios15050317 (PMC12109915; doi:10.3390/bios15050317)
Supplement: Supplementary file 1 [file biosensors-15-00317-s001.zip › Supplementary Files/Biosensors-SI.docx]

**Supporting Information**

**One-Pot Detection of miRNA by Dual Rolling Circle Amplification at Ambient Temperature with High Specificity and Sensitivity**

Wenhua Sun ^1^, Kunling Hu ^1†^, Ziting Song ^1^, Ran An ^1,2,*^ and Xingguo Liang ^1,2,*^

^1^ State Key Laboratory of Marine Food Processing & Safety Control, College of Food Science and Engineering, Ocean University of China, No. 1299 Sansha Road, Qingdao 266404, China; sunwenhua4988@stu.ouc.edu.cn (W.S.); klhu1@gzu.edu.cn (K.H.); szt@stu.ouc.edu.cn (Z. S.)

^2^ Laboratory for Marine Drugs and Bioproducts, Qingdao Marine Science and Technology Center, No. 1 Wenhai Road, Qingdao 266237, China

**^*^** Correspondence: ar@ouc.edu.cn (R.A.); liangxg@ouc.edu.cn (X.L.)

† Current address: College of life sciences, Guizhou University, Huaxi District, Guiyang City, Guizhou, 550025, China

**Table of Contents:**

[Table S1 The sequences of DNA and RNA used in this study.	3](#_Toc197333665)

[Figure S1 Circularization results of templates	4](#_Toc197333666)

[Figure S2 Real time record of PG-RCA with splint-aided circularization	5](#_Toc197333667)

[Figure S3 Effect of C2 on non-specific amplification	6](#_Toc197333668)

[Figure S4 Effect of the 3’→5’ exonuclease activity of phi29 on amplification	7](#_Toc197333669)

[Figure S5 Sequence design for detecting miR-106 with dual RCA	8](#_Toc197333670)

[Figure S6 Specificity evaluation of dual RCA (miR-155)	9](#_Toc197333671)

[Figure S7 Specificity evaluation of dual RCA (miR-106)	10](#_Toc197333672)

[Figure S8 Determination of miRNA in 2% human serum	11](#_Toc197333673)

Table S1 The sequences of DNA and RNA used in this study.

| Name | Sequences (5′→3′)^b)^ | Length (nt) |
| --- | --- | --- |
| C1-155 ^a)^ | CGTGTGAAACATCCTTGAG*AATTCATTGAACTATATCTACACCCCTATCACGATTAGCATTAACACACGACCGAAGGT | 78 |
| C2-155 ^a)^ | CTGACGTCATATATGCATTGAACTATATCTACACCGTTACTAATCGTCAGACCGAAGGT | 59 |
| C1-106 ^a)^ | CGTGTGTTTGATCCTTGTG*AATTCTCTTGACGACTTGAACCTACCTGCACTGTAAGCACTTTTCACACGACCGAAGGT | 78 |
| C2-106 ^a)^ | CGTAGCTACATAATTCTCTTGACGACTTGAACCTAGTTACTAATGCTACGACCGAAGGT | 59 |
| miR-155 | UUAAUGCUAAUCGUGAUAGGGGU | 23 |
| mis1^c)^ | UUAAUGCUAAUCCUGAUAGGGGU | 23 |
| mis-2^c)^ | UUAAUGCUAAUCCUGAUAAGGGU | 23 |
| mis-3^c)^ | UUAAUACUAAUCCUGAUAAGGGU | 23 |
| miR-106 | AAAAGUGCUUACAGUGCAGGUAG | 23 |
| miR-159 | UUUGGAUUGAAGGGAGCUCUA | 21 |
| miR-20 | UAAAGUGCUUAUAGUGCAGGUAG | 23 |

^a)^ C1-155, C2-155, C1-106, C2-106 were phosphorylated before ligation. ^b)^ In C1-155 and C1-106, “*” represents phosphorothioate modification, the underlined part represents the recognition sequence of EcoRI-HF. ^c)^ The nucleotide in red font represents that it is mutated (different from the sequence of original miR-155).

Figure S1

**Figure S1.** Circularization results of templates. (A) Circularization results of template 1. (B) Circularization results of template 2. Lane 1, Linear template. Lane 2, Linear template treated with T4 Dnl. Lane 3, the products in Lane 2 were further treated with Exonuclease I and Exonuclease III to remove linear substrate and polymers. Conditions: 5 μM linear ssDNA, 0.5 U/μL T4 Dnl, 0.1× T4 ligase buffer, 25°C, 12 h. Analyzed by 12% dPAGE.

Figure S2

**Figure S2.** Real time record of PG-RCA at various concentrations of miR-155 using the circular ssDNA prepared with splint-aided circularization. PG-RCA conditions: 0.1 nM C1, 25 U/mL phi29, 50 U/mL EcoRI-HF, 1× SYBR Green II, 0.5× phi29 buffer, 0.5× rCutsmart buffer, 400 μM each dNTP, and incubation at 37°C. The sensitivity is much lower than that for splint-free circularization.

Figure S3

**Figure S3.** Effect of C2 on non-specific amplification. The dark green bars represent the fluorescence signal for the 1 pM target group, while the light green bars correspond to those for the negative control group. Other conditions: 0.1 nM C1, 1 pM Pr, 25 U/mL phi29, 50 U/mL EcoRI-HF, 1× SYBR Green II, 0.5× phi 29 buffer, 0.5× rCutsmart buffer, 400 μM each dNTP, and incubation at 37°C for 120 min.

Figure S4

**Figure S4.** Effect of the 3’→5’ exonuclease activity of phi29 on amplification. The orange line represents the amplification of the primer with phosphorothioate modifications at the 3’ end (PS-Pr). The green line represents the amplification of the normal primer without modification (Pr). Conditions: 0.1 nM C1, 10 nM C2, 100 fM Pr, 25 U/mL phi29, 50 U/mL EcoRI-HF, 1× SYBR Green II, 0.5× phi 29 buffer, 0.5× rCutsmart buffer, 400 μM each dNTP, and incubation at 37°C for 150 min.

Figure S5

**Figure S5.** Sequence design for detecting miR-106 with dual RCA. The phosphate bond between G and A in the recognition site (GAATTC) of EcoRI-HF (a restriction enzyme) is replaced by phosphorothioate to avoid the cleavage of C1 (78 nt). C2 (59 nt) has no modification.

Figure S6

**Figure S6.** Specificity evaluation of dual RCA (miR-155). (A) Real time record of different miRNAs. Pr(-): denotes the absence of miRNA, miR-155(-) indicates the presence of 1 pM miR-20, miR-106, and miR-159, but no target miR-155, and “mixture” refers to a reaction containing all miRNAs. (B) Real time record of miR-155 with different numbers of mismatches. Pr(-) denotes the absence of miRNA, miR-155(-) indicates the presence of 1 pM of mis1, mis2, and mis3, but no target miR-155, and “mixture” refers to a reaction containing all the miRNAs. Other conditions: 0.1 nM C1, 10 nM C2, 1.0 pM miRNA, 25 U/mL phi29, 50 U/mL EcoRI-HF, 1× SYBR Green II, 0.5× phi 29 buffer, 0.5× rCutsmart buffer, 400 μM each dNTP, and incubation at 37°C for 120 min.

Figure S7

**Figure S7.** Specificity evaluation of dual RCA (miR-106). (A) Real time record of different miRNAs. Pr(-): denotes the absence of miRNA, miR-106(-) indicates the presence of 1 pM miR-20, miR-155, and miR-159, but no target miR-106, and “mix” refers to a reaction containing all miRNAs. (B) Fluorescence intensity for targeting miR-106 in the presence of various other miRNAs. The reaction conditions: 0.1 nM C1, 10 nM C2, 1 pM different miRNA, 25 U/mL phi29, 50 U/mL EcoRI-HF, 1× SYBR Green II, 0.5× phi 29 buffer, 0.5× rCutsmart buffer, 400 μM each dNTP, and incubation at 37°C for 100 min.

Figure S8

**Figure S8.** Determination of miRNA in 2% human serum. (A) Real time record of various concentrations of target miR-155 in 2% serum samples. (B) Calibration curve showing the linear relationship between the threshold time (Tt) and the logarithmic concentration (lgM) of miR-155, within the range of 10^−13^ to 10^−10^ M. The reaction conditions: 0.1 nM C1, 10 nM C2, different concentrations miR-155, 25 U/mL phi29, 50 U/mL EcoRI-HF, 1× SYBR Green II, 0.5× phi 29 buffer, 0.5× rCutsmart buffer, 400 μM each dNTP, and incubation at 37°C.
